# Informal Caregivers’ Roles in Dementia: The Impact on Their Quality of Life

**DOI:** 10.3390/life10110251

**Published:** 2020-10-23

**Authors:** Cindy E. Frias, Esther Cabrera, Adelaida Zabalegui

**Affiliations:** 1 Mental Health Nurse, Hospital Clinic, 08036 Barcelona, Spain; cfrias@clinic.cat; 2 School of Health Sciences, University of Barcelona, 08036 Barcelona, Spain; 3 School of Health Sciences, TecnoCampus Universitat, Pompeu Fabra, 08301 Mataró, Spain; ecabrera@tecnocampus.cat; 4 Department of Care Management and Social Work, Sechenov University, Moscow 101000, Russia; 5 Department of Nursing, Hospital Clínic de Barcelona, 08001 Barcelona, Spain

**Keywords:** dementia, informal caregivers, quality of life, emotional wellbeing, nurse

## Abstract

The constantly changing process of caring for a person with dementia affects the informal caregivers’ role due to its psychosocial impact. This cross-sectional study aimed to analyze the impact of the Person with Dementia informal caregiver’s role caregiver to a person with dementia on the self-perceived quality of life (QoL) of the caregiver. In total, 160 informal caregivers were recruited between January and December 2019. Informal caregivers’ quality of life was assessed using the European Quality of Life 5-Dimension scale, burden with the Zarit Burden Scale, emotional wellbeing using the General Health Questionnaire, and caregiver reactions using Caregiver Reaction Aspects. Patients’ cognitive impairment was assessed with the Mini-Mental State Examination, their quality of life using Quality of Life in Alzheimer’s Disease, and neuropsychiatric symptoms using the Neuropsychiatric Inventory. Outcomes were studied using the Pearson correlation coefficient and ANOVA test. Most informal caregivers’ outcomes were significantly associated with their quality of life. Male informal caregivers have a slightly better quality of life than female caregivers (*p* < 0.001). Caregiver burden (*p* < 0.001), psychological wellbeing (*p* < 0.001) and negative aspects of caregiving on health (*p* < 0.001) correlated moderately with informal caregivers’ quality of life. Factors associated with dementia, including the course of the illness and its severity with the presence of neuropsychiatric symptoms can negatively affect the informal caregiver’s role and produce a low self-perception of quality of life; thus, social and professional support for informal caregivers is essential.

## 1. Introduction

The progressive decrease in the cognitive and functional abilities of a person with dementia (PwD) and the presence of neuropsychiatric symptoms (NS) lead to behavioral changes which interfere with the patient’s autonomy and daily functioning [1]. Dementia is known to affect around 10 million people in Europe, of which 800,000 cases are people diagnosed in Spain, with more than 86,000 of these in Catalonia [2,3]. The adjustments for daily activities generate a change in the roles in a family, so that a member becomes an informal caregiver (IC), usually the spouse or one of the offspring [4]. It is estimated that about 84% of PwD live in their own home and it is the caregiver who is responsible for keeping them in optimal health; this role can extend up to an average of 6.5 years [4,5,6].

As the illness is progressive, the process of caring for a PwD changes constantly and has a significant psychosocial impact on the family and the PwD. Due to the considerable stress involved, it is essential to prevent the physical and mental effects on the IC, which include cardiovascular disease, stress-related disorders, burden, sleep problems, anxiety, and even depression, all of which affect the informal caregivers’ role [6]. In fact, there is strong evidence that the feeling of burden among IC of PwD is greater than in other types of care and that this frequently leads to them working fewer hours or losing their job, with a resulting decrease in their own quality of life (QoL) [4,5,6]. Some studies show that the emotional effects of burden can also have a negative impact on the PwD’s life, which can be even greater than the degree of severity of the disease itself [7,8]. QoL is a multidimensional concept, which varies between individuals and depends directly on the life stage of the person, and on positive and negative factors related to the changes that occur at each stage [6,8]. De Miguel et al. point out that QoL is a concept that is difficult to define and that this has a special connotation when associated with dementia care. The same authors report that a caregiver having a better or worse QoL is directly related to the presence of neuropsychiatric symptoms in the PwD. They conclude that intervening to decrease or control NS and depressive symptoms in the PwD will improve or maintain informal caregivers’ QoL [9]. Nevertheless, caring generates positive aspects with respect to ICs’ satisfaction with the work carried out, the ability to overcome certain difficulties and the strengthening of the bond with the person receiving care [5]. Under these circumstances, home care needs to be oriented towards improving ICs’ roles and to improving the self-perceived caregiver’s QoL, reducing burden, and promoting home management strategies [5,7]. In this sense, professional support and counselling allow for the early detection and prevention of alterations in the IC’s physical and mental health [5]. The aim of this study was to analyze the impact of the role of an informal caregiver to a PwD on the self-perceived QoL of the caregiver. 

## 2. Materials and Methods 

### 2.1. Study Design

This was a cross-sectional study based on data from a larger investigation.

### 2.2. Study Settings and Participants

The study was conducted with informal caregivers (IC) of a person with dementia (PwD). Participants were recruited in 3 primary care centers in Barcelona. During the PwD’s regular follow-up visits with the family physician (FP) or nurse, ICs were invited to participate in the study. Those who accepted were contacted by telephone by a person from the research team.

During primary care visits, the family physician (FP) or nurse provided additional information about the study verbally or through pamphlets. Those participants who met inclusion criteria received a second call to make an appointment at home for the collection of initial data. The interviews were conducted between January and December 2019. All participating ICs and PwDs provided signed informed consent. 

The inclusion criteria for the informal/family caregivers, defined as people who provided informal care on regular basis, were as follows: (1) > 18 years old; (2) living with the person with dementia or visiting him/her at least twice a week; (3) not being paid for caregiving. The inclusion criteria for the people with dementia were as follows: (1) having a Mini-Mental State Examination (MMSE) test score < 24; (2) living at home. The exclusion criteria for the PwD were as follows: (1) having another psychiatric illnesses or Korsakov’s syndrome. PwD were informed about the study and they provided signed informed consent at home. In the case of the severe cognitive impairment of the PwD, consent was only signed by the IC.

The complete study was revised and approved prior to study initiation by the Ethical Committee at the Clinic Hospital (Reg. HCB/2018/0737) and followed the recommendations of the Declaration of Helsinki.

### 2.3. Data Collection

Data were collected during face-to-face interviews with by trained interviewers following instructions in a written manual. IC and PwD were the informants in the home care setting. Interviews were carried out between January and December 2019. Interviewers were nurses with clinical experience and at least a master’s degree. Furthermore, they received additional training on data collection, including all procedures, the content of the assessments and the completion of questionnaires.

### 2.4. Measures and Instruments

Socio-demographic and other potential variables related to QoL in IC and PwD were collected. 

#### 2.4.1. Informal Caregiver Assessments

Caregivers’ QoL was assessed using the European Quality of Life 5-Dimension (EQ-5D) scale, a measure of self-reported quality of life that is applicable to a wide range of health conditions and treatments. EQ-5D has two components: a health state description and self-reported evaluation. The first component consists of 5 single-item dimensions, including mobility, self-care, usual activities, pain/discomfort, and anxiety/depression. Each dimension has a 3-point response scale designed to indicate the degree of the problem. The index of health states generated by the EQ-5D is obtained using a composite time trade-off (cTTO) valuation technique proposed for the study in the Spanish general population. The index ranges from 1 (the best health status) to 0 (death). A visual analogue scale is the second component of the questionnaire, asking respondents to mark their health status on the day of the interview on a 20-cm vertical scale with end points of 0 and 100. This scale includes notes at the ends of the scale, explaining that the bottom rate (0) corresponds to “the worst health you can imagine”, and the highest rate (100) corresponds to “the best health you can imagine” [10]. 

Burden was assessed using the Spanish version of the Zarit Burden Scale (V1, Navarra, Spain). This evaluated subjective perception of care, emotional and physical burden, financial difficulties and limitations to social activities associated with care provision. It consists of 22 items on a 5-point Likert-type scale ranging from 0 = “never” to 4 = “almost always”, with higher scores indicating a high degree of burden. The scale has excellent internal consistency with a Cronbach’s alpha of 0.92 [11]. Emotional wellbeing was assessed using the General Health Questionnaire (GHQ-12). As a unidimensional measure, it seems to be a useful screening tool for the assessment of mental distress or a minor psychiatric morbidity in contexts such as primary care or in the general population. It has 12 items—6 positively worded and 6 negatively worded. It is a 4-point Likert-type scale (0–1–2–3) that can be transformed into a dichotomous score (0–1) called the GHQ score. The GHQ-12 demonstrates good reliability in studies carried out with a Cronbach’s alpha between 0.82 and 0.86 [12,13].

Caregiver reactions were evaluated using Caregiver Reaction Aspects (CRA). This is an instrument designed to assess specific facets of caregiving, including both negative and positive dimensions of caregiving reactions. It consists of 24 items in five subscales: self-esteem (score range 7–35), lack of family support (range 5–25), financial problems (range 3–15), disrupted schedule (range 5–25) and health problems (range 4–20). Responses are represented on a Likert-type scale from 1 = strongly disagree to 5 = strongly agree. A higher score on the caregiver’s self-esteem subscale indicates a more positive reaction to caregiving, while higher scores on the other four subscales indicate greater negative reactions. Reliability analyses showed that standardized Cronbach’s alphas varied between 0.62 and 0.83 for the separate subscales, indicating sufficient internal consistencies. Construct validity was supported. The CRA proves to be a feasible, reliable, valid instrument for assessing both negative and positive reactions to caregiving [14,15].

#### 2.4.2. PwD Assessments

The degree of cognitive impairment was assessed with the Mini-Mental State Examination (MMSE) and the Global Deterioration Scale (GDS), where higher scores indicate less cognitive impairment [16,17]. 

QoL in PwD was also evaluated using the Quality of Life in Alzheimer’s Disease (QoL-AD) instrument. The QoL-AD consists of 13 items relating to QoL, each measured on a four-point scale (ranging from 1 = poor to 4 = excellent). Possible scores on the QoL-AD range from 13 to 52. Higher scores indicate better QoL [18]. Neuropsychiatric symptoms in the PwD were measured using the Neuropsychiatric Inventory (NPI), which includes 10 behavioral domains (delusions, hallucinations, dysphoria, anxiety, agitation/aggression, euphoria, disinhibition, irritability, apathy and aberrant motor activity) with 7–8 sub-questions and measures severity (3-point scale) and frequency (4-point scale). The total NPI score is the sum of the subscale scores. Higher scores indicate more behavioral disturbance. Analyses showed that the standardized Cronbach’s alpha is 0.783 [19]. 

Measurement instruments were selected based on their psychometric properties (validity, reliability), clinical utility and suitability for the target settings and population.

### 2.5. Statistical Analyses

Descriptive data are presented as the mean and standard deviation (SD) for continuous outcomes and number and the percentage (%) for categorical outcomes. The association between the informal caregivers’ quality of life (EQ-5D and VAS), PwD outcomes (age, gender, type of dementia, time since diagnosis, illness severity, ability to perform activities of daily living, comorbidity, behavioral disturbance, quality of life, cognitive function) and IC outcomes (age, gender, marital status, relationship with patient, living with patient, caregiver burden, psychological wellbeing, social support, preparedness for caregiving, care needs of families, caregiving competence, positive and negative aspects of caregiving) was studied using the Pearson correlation coefficient for continuous outcomes and the ANOVA test for categorical outcomes. Outcomes with a *p*-value < 0.05 were included in a multiple linear regression model to determine outcomes independently associated with the informal caregivers’ quality of life. The stepwise Akaike Information Criterion (AIC) method was used for variable selection [19]. Variance inflation factor (VIF) was used to quantify the severity of the multicollinearity effect. VIF values higher than 5 are considered to have high multicollinearity [20]. All significance tests were 2-tailed, and values of *p* < 0.05 were considered significant. Statistical analyses were conducted using the R 3.6.1 (Vienna, Austria) for Windows statistical software package for Windows [21,22].

## 3. Results

### 3.1. Sociodemographic Characteristics and Outcomes

A total of 160 informal caregivers of PwD were included in the study. The sociodemographic characteristics and outcomes of informal caregivers and persons with dementia are detailed in Table 1 and Table 2. The mean age was 63.7 (SD 12.8) years for IC and 79.1 (SD 8.2) years for PwD. In total, 76.2% of IC and 54.4% of PwD were female. Almost half of IC (51.2%) were spouses and 75.6% of IC were living with the PwD. Regarding informal caregivers, 119 (76.8%) were considered to have a moderate to high burden due to caregiving. Overall, 50 (32.3%) caregivers indicated poor psychological wellbeing and 33 (21.3%) low social support (Table 1). The main type of dementia was Alzheimer’s (70%). Eighty-five (62.1%) PwD had moderate or severe disease severity. In total, 103 (68.2%) had some degree of disability (Table 2). 

### 3.2. Informal Caregivers’ QoL

The QoL of informal caregivers of female persons with dementia was slightly better than the QoL of caregivers of male persons with dementia (0.72 female patient versus 0.65 male patient, *p* = 0.002) (Table 3). The severity of PwDs’ behavioral disturbance was another outcome associated with informal caregivers’ QoL even though the correlation is low (r = −0.178, *p* = 0.028). Self-perceived QoL was associated with time since diagnosis and PwD QoL measured by QoL-AD. Less time since diagnosis (r = −0.230, *p* = 0.009) and better PwD QoL (r = 0.165, *p* = 0.042) indicate better informal caregiver QoL. Despite the tendencies previously mentioned, the strength of the relationships between the informal caregivers’ QoL and PwD outcomes was low. Most of the caregivers’ outcomes were significantly associated with their QoL (Table 4). Male caregivers have a slightly better QoL than female caregivers (0.77 male caregiver versus 0.67 female caregiver, *p* < 0.001). Caregiver burden (r = −0.437, *p* < 0.001), psychological wellbeing (r = −0.474, *p* < 0.001) and impact of negative aspects of caregiving on health (r = −0.465, *p* < 0.001) were moderately correlated with informal caregivers’ QoL. In the case of self-perceived quality of life, impact of negative aspects of caregiving on health (r = −0.439, *p* < 0.001), psychological wellbeing (r = −0.370, *p* < 0.001) and caregiver self-esteem (r = 0.317, *p* < 0.001) were the three outcomes with the highest correlation with self-perceived QoL. Moreover, the results show that caregivers with low social support (<33 on the DUKE scale) have significantly lower QoL compared to those with normal social support (≥33 on the DUKE scale) (63.79 low social support versus 73.18 normal social support, *p* = 0.003).

### 3.3. Outcomes Independently Associated with the Informal Caregivers’ QoL

The results of the multiple linear regression models are detailed in Table 5. There is no multicollinearity (the VIF are lower than 5). After including PwDs’ and informal caregivers’ outcomes significantly associated with QoL in the model, patient gender, caregiver burden and the impact of negative aspects of caregiving on health appeared as the only factors independently associated with QoL. On the other hand, time since diagnosis, informal caregiver’s psychological wellbeing and impact of negative aspects of caregiving on health were the factors independently associated with self-perceived quality of life. Both models were able to explain around 30% of the total variability for quality of life, indicating that other important variables associated with QoL exist that are not related to informal caregivers’ or PwDs’ outcomes.

## 4. Discussion

Dementia has important consequences for the quality of life of informal caregivers. The current study analyzes the impact of the role of informal caregivers to PwD on the self-perceived QoL of caregivers. Findings confirm that the progressive nature of dementia, burden, and aspects related to informal caregivers’ physical and mental health increase the vulnerability of informal caregivers. This has direct repercussions on care provision in the home, which are associated with self-perceived QoL. 

IC and PwDs’ sociodemographic characteristics are similar to those of other studies. The IC is, in general, a person over 60 years old, who is female, and who lives with and shares a bond with the PwD—in most cases, the spouse of the PwD [23,24]. For their part, the PwD are frequently female with an average age of over 70 years. As such, our findings indicate that gender is one of the variables influencing perception of QoL in both IC and PwD. Alzheimer’s is the most frequent type of dementia with a manifestation of neuropsychiatric symptoms, which, with the passing of time, entail a need for continuous care. This can lead to a certain degree of burden and to the appearance of alterations in the emotional wellbeing of the IC of a depressive or anxious type, and/or psychological distress. However, our results show that time since diagnosis is also one of the associated factors that influences self-perceived QoL, so that—in the initial or intermediate phases of dementia, when the PwD demand greater attention due to the progress of the illness—there is a need for new interventions that can slow down or arrest this progression and maintain or improve PwDs’ results [2,25].

### 4.1. Informal Caregivers’ Quality of Life 

Different perspectives have been unified over the years to help to define and analyze the concept of QoL and its relationship with care. Nevertheless, its multidimensional, nebulous nature defies a precise description of its defining variables. Thus, assessments of physical and emotional wellbeing are important in the perception of better or worse QoL in the informal caregiver [8,25,26]. 

Regarding the relationship between QoL and the role of the informal caregiver, it would appear that different aspects of dementia positively or negatively influence the perception of better or worse QoL. One of the main aspects that affects the informal caregiver’s role is illness severity, one of the principal reasons for role reorganization, as PwD care-related activities change over the years with a possible need to increase the number of hours of daily care [25]. Kaizik et al. stated that the severity of dementia is a factor which influences the appearance of caregiver burden as those IC who dedicate more time to general supervision and provide more assistance to PwD in daily activities experience a high level of tension. Study findings demonstrate that more than 70% of ICs have a high degree of burden, with low levels of emotional wellbeing as a result of their informal caregiving role and a greater number of hours dedicated to care provision. Comparative studies between different types of caregivers show that the IC of PwD report a significantly higher degree of burden than other types of caregivers due to the greater number of hours per day devoted to patient care and that, in addition, there is a strong association between high levels of depression and advanced states of dementia [26]. Therefore, identification of those more specific needs is useful for the intervention of professionals who guide home care [25,27,28]. 

Another aspect of dementia associated with informal caregiver QoL is the presence of neuropsychiatric symptoms (NS). In fact, the appearance of these symptoms in PwD in the sample negatively influenced the QoL of the IC. In accordance with Karg et al., the presence and severity of NS, together with the age at onset and the number of years of providing care, affect the personal life and role of the IC as they increase the PwD and ICs’ needs and this increase is possibly perceived as a reduction in QoL itself [25,26,29,30]. This has led to an analysis of the impact of the informal caregiver’s role on their own lives in order to be able to intervene early in aspects that can be modified to delay or avoid the emergence of alterations in their physical or emotional wellbeing. Some reviews suggest that the positive and negative dimensions of care are different concepts, although there are an increasing number of studies that show the association between these dimensions and the appearance of burden and these alterations in the IC [31]. Indeed, in the present study, the impact of the negative aspects of care on health is one of the variables with the greatest correlation with the self-perception of QoL. Acosta et al. reported that the informal caregiver role can, over time, negatively affect both the personal and working life, as daily care provided over many years means less time for leisure activities and self-care and can even lead to conflict within the family [5,28]. 

Finally, our findings highlight the significant association between low social support, whether from family and/or friends, and self-perceived QoL as it appears that those ICs with low social support have a significantly lower QoL than those with good social support. This is consistent with other studies which indicate that IC with good social support experience fewer alterations in their emotional wellbeing and less stress as they have more time to enjoy pleasant activities. This would represent an effective strategy for the prevention of alterations in the mental health of IC and an improvement in self-perceived QoL since, according to the authors, it would be based on the fact that “social support does not only directly affect depression but also exercises an indirect influence on depression through the informal caregiver role”. These findings also emphasize the need for comprehensive support services for both PwD and IC [32,33]. 

### 4.2. Limitations 

Despite the relevant aspects of our study and the correlation of variables associated with the QoL of PwD and IC, some limitations should be recognized. First, convenience sampling was performed, which could limit the generalization of the results. However, variables related to the PwD and IC were identified and considered in the analysis of causal relationships. Second, socioeconomic differences between participants were not taken into account and it would have been useful to analyze economic impact on caregivers’ QoL.

## 5. Conclusions

The present study offers a description of the relationship between the informal caregiver role and the QoL of informal caregivers of people with dementia. It can be observed that factors associated with dementia such as its progressive nature and severity with the presence of neuropsychiatric symptoms can have a negative influence on the role of the informal caregiver, leading to the emergence of burden and a consequent self-perception of low QoL. For this reason, social and professional support is essential for IC to facilitate care provision and improve the quality of life of IC and PwD. Similarly, follow-up and accompaniment of informal caregivers and persons with dementia by health professionals can promote timely detection of the appearance of alterations in physical health and emotional wellbeing, allowing for early intervention and referrals to specific centers. 

## Figures and Tables

**Table 1:** Informal caregivers’ characteristics and outcomes.

Variable	Total (n = 160)
Age, years	63.7 ± 12.8
Gender, female	122 (76.2)
Marital status caregiver	
Married/cohabitant	127 (79.4)
Never married	13 (8.1)
Divorced	10 (6.2)
Widowed	4 (2.5)
No response	6 (3.8)
Relationship with person with dementia	
Spouses	82 (51.2)
Offspring	61 (38.1)
Others	17 (10.7)
Caregiver lives with patient	121 (75.6)
Caregiver burden (ZBI)	33.3 ± 15.3
Little to mild burden (0–20)	36 (23.2)
Mild to moderate burden (21–40)	70 (45.2)
High burden (>40)	49 (31.6)
Quality of Life (EQ-5D)	0.7 ± 0.2
Self-reported quality of life (VAS)	71.2 ± 17.2
Psychological well-being (GHQ-12)	3.6 ± 3.3
Normal (0–4)	105 (67.7)
Poor (5–12)	50 (32.3)
Social support (Duke)	40.8 ± 9.6
Low (<33)	33 (21.3)
Normal (33–55)	122 (78.7)
Care needs of families (FIN)	
Importance	35.9 ± 5.4
Needs are important (36–44)	67 (43.5)
Needs are not important (<36)	87 (56.5)
Fulfillment	24.0 ± 10.2
Needs are fulfilled (26–44)	71 (46.1)
Needs are not fulfilled (<26)	83 (53.9)
Total	59.9 ± 12.1
Needs are identified (65–88)	63 (40.9)
Needs are not identified (<65)	91 (59.1)
Positive and negative aspects of caregiving (CRA)	
Caregiver self-esteem	27.6 ± 4.2
Lack of family support	12.7 ± 4.3
Impact on finances	8.0 ± 2.3
Impact on schedule	16.5 ± 4.9
Impact on health	10.7 ± 4.4

Data are presented as number (percentage) or means ± standard deviation. ZBI: Zarit Burden Interviews, EQ-5D: EuroQol 5 Dimension, VAS: Visual Analogue Scale, GHQ-12: General Health Questionire 12 item version, FCI: Family Caregiving Inventory, FIN: Family Inventory of Needs, PCSS: Pearlin Caregivers’ Stress Scale, KESO: Knowledge Expectations of significant other-scale, RKSO: Received Knowledge of significant other-scale, CRA: Caregiver Reaction Assessment.

**Table 2:** People with dementia characteristics and outcomes.

Variable	Total (n = 160)
Age, years	79.1 ± 8.2
Gender, female	87 (54.4)
Type of dementia	
Alzheimer	112 (70.0)
Unknown	21 (13.1)
Vascular dementia	11 (6.9)
Others	9 (5.6)
Dementia with Lewy bodies	6 (3.8)
Alzheimer’s with cerebrovascular disease	1 (0.6)
Time since diagnosis, years	5.4 ± 3.2
Disease severity (MMSE)	17.3 ± 6.6
Mild (21–28)	52 (38.0)
Moderate (15–20)	53 (38.7)
Moderately severe/severe (<15)	32 (23.4)
Dependency in ADL (Katz Index)	3.9 ± 2.1
Disability (0–2)	45 (29.8)
Partial disability (3–5)	58 (38.4)
Functional independence (6)	48 (31.8)
Comorbidity (Charlson Index)	1.5 ± 1.2
0	28 (17.5)
1	79 (49.4)
2	23 (14.4)
More than 2	30 (18.8)
Behavioral disturbance (NPI)	
Severity	6.7 ± 5.4
Distress	4.0 ± 6.1
Quality of Life	
EQ-5D	0.6 ± 0.3
Self reported (VAS)	67.3 ± 20.1
QoL-AD	29.3 ± 5.1
Cognitive function (GDS)	
Very mild cognitive decline	2 (1.2)
Mild cognitive decline	34 (21.2)
Moderate cognitive decline	69 (43.1)
Moderately severe cognitive decline	31 (19.4)
Severe cognitive decline	16 (10.0)
Very severe cognitive decline	8 (5.0)

Data are presented as number (percentage) or means ± standard deviation. MMSE: Mini-Mental Stage Examination, ADL: activities of daily living, NPI: Neuropsychiatric Inventory, EQ-5D: EuroQol 5 Dimension, VAS: Visual Analogue Scale, QoL-AD: Quality of Life in Alzheimer’s Disease, GDS: Global Deterioration Scale.

**Table 3:** Association between people with dementia outcomes and informal caregivers’ quality of life.

Variable	Quality of Life (EQ-5D)	*p*-Value ^†^	Self-Reported Quality of Life (VAS)	*p*-Value ^‡^
Age, years	−0.109	0.197	−0.054	0.522
Gender		**0.002**		0.933
Male	0.65 (0.15)		71.03 (18.50)	
Female	0.72 (0.15)		71.27 (16.08)	
Type of dementia		0.181		0.429
Alzheimer’s	0.70 (0.15)		70.57 (17.93)	
Unknown	0.65 (0.15)		75.00 (15.34)	
Vascular dementia	0.62 (0.14)		73.18 (12.10)	
Others	0.73 (0.05)		68.89 (18.50)	
Dementia with Lewy bodies	0.65 (0.24)		75.00 (12.25)	
Time since diagnosis, years	−0.050	0.573	−0.230	**0.009**
Disease severity (MMSE)	−0.120	0.166	0.090	0.299
Mild (21–28)	0.67 (0.15)		71.86 (18.05)	
Moderate (15–20)	0.72 (0.14)		72.96 (14.26)	
Moderately severe/severe (<15)	0.70 (0.15)		67.42 (18.48)	
Dependency in ADL (Katz Index)	−0.023	0.781	0.028	0.731
Disability (0–2)	0.71 (0.18)		71.16 (18.19)	
Partial disability (3–5)	0.66 (0.15)		71.46 (16.08)	
Functional independence (6)	0.71 (0.13)		70.68 (17.62)	
Comorbidity (Charlson Index)	−0.096	0.237	0.045	0.585
0	0.72 (0.14)		70.04 (20.05)	
1	0.68 (0.15)		71.81 (16.34)	
2	0.70 (0.19)		67.70 (22.78)	
More than 2	0.67 (0.14)		73.10 (11.05)	
Behavioral disturbance (NPI)				
Severity	−0.178	**0.028**	−0.029	0.723
Distress	−0.053	0.517	−0.100	0.218
Quality of Life				
EQ-5D	0.031	0.753	0.105	0.286
Self-reported (VAS)	0.049	0.638	0.193	0.060
QoL-AD	0.116	0.153	0.165	**0.042**
Cognitive function (GDS)		0.236		0.537
Very mild cognitive decline	0.66 (0.10)		74.50 (34.65)	
Mild cognitive decline	0.63 (0.16)		72.12 (18.53)	
Moderate cognitive decline	0.71 (0.14)		70.28 (16.24)	
Moderately severe cognitive decline	0.71 (0.16)		75.39 (15.15)	
Severe cognitive decline	0.67 (0.20)		65.67 (23.06)	
Very severe cognitive decline	0.74 (0.12)		67.50 (8.86)	

Data are presented as means (standard deviation) or Pearson correlation coefficient. In bold, people with dementia outcomes significantly (*p* < 0.05) associated with informal caregivers’ quality of life. ^†^ Association between people with dementia outcomes and EQ-5D. ANOVA test was used for categorical outcomes and correlation test for continuous outcomes. ^‡^ Association between people with dementia outcomes and VAS. ANOVA test was used for categorical outcomes and correlation test for continuous outcomes. MMSE: Mini-Mental State Examination, ADL: activities of daily living, NPI: Neuropsychiatric Inventory, EQ-5D: European Quality of Life 5-Dimension scale, VAS: Visual Analogue Scale, QoL-AD: Quality of Life in Alzheimer’s Disease, GDS: Global Deterioration Scale.

**Table 4:** Informal caregivers’ outcomes associated with informal caregivers’ quality of life.

Variable	Quality of Life (EQ-5D)	*p*-Value ^†^	Self-Reported Quality of Life (VAS)	*p*-Value ^‡^
Age, years	−0.042	0.603	−0.051	0.531
Gender		**<0.001**		0.204
Male	0.77 (0.09)		74.55 (12.95)	
Female	0.67 (0.16)		70.23 (18.17)	
Marital status caregiver		0.936		0.479
Married/cohabitant	0.69 (0.15)		71.19 (17.81)	
Never married	0.69 (0.16)		72.31 (17.98)	
Divorced	0.65 (0.20)		72.50 (5.89)	
Widowed	0.71 (0.03)		57.50 (9.57)	
No response	0.74 (0.08)		82.50 (17.68)	
Relationship with person with dementia		0.790		0.829
Spouses	0.68 (0.15)		71.94 (17.90)	
Offspring	0.70 (0.16)		70.43 (15.62)	
Others	0.69 (0.18)		69.64 (19.95)	
Caregiver lives with patient		0.249		0.971
No	0.72 (0.15)		71.06 (14.88)	
Yes	0.68 (0.15)		71.18 (17.81)	
Caregiver burden (ZBI)	−0.437	**<0.001**	−0.204	**0.011**
Little to mild burden (0–20)	0.75 (0.09)		74.64 (17.11)	
Mild to moderate burden (21–40)	0.71 (0.12)		73.28 (14.98)	
High burden (>40)	0.61 (0.20)		65.65 (18.99)	
Psychological wellbeing (GHQ-12)	−0.474	**<0.001**	−0.370	**<0.001**
Normal (0–4)	0.73 (0.12)		74.66 (15.15)	
Poor (5–12)	0.62 (0.18)		63.71 (18.92)	
Social support (Duke)	0.148	0.067	0.240	**0.003**
Low (<33)	0.66 (0.20)		63.79 (19.80)	
Normal (33–55)	0.70 (0.14)		73.18 (15.87)	
Care needs of families (FIN)				
Importance	0.003	0.970	−0.062	0.446
Needs are important (36–44)	0.69 (0.16)		69.23 (18.82)	
Needs are not important (<36)	0.69 (0.15)		72.23 (15.78)	
Fulfillment	0.191	**0.019**	0.182	**0.026**
Needs are fulfilled (26–44)	0.73 (0.13)		73.22 (15.55)	
Needs are not fulfilled (<26)	0.65 (0.16)		69.02 (18.28)	
Total	0.162	**0.048**	0.124	0.129
Needs are identified (65–88)	0.70 (0.14)		71.59 (16.22)	
Needs are not identified (<65)	0.68 (0.16)		70.50 (17.84)	
Positive and negative aspects of caregiving (CRA)				
Caregiver self-esteem	0.254	**0.002**	0.317	**<0.001**
Lack of family support	−0.098	0.239	−0.092	0.267
Impact on finances	−0.233	**0.004**	−0.255	**0.002**
Impact on schedule	−0.262	**0.001**	−0.224	**0.007**
Impact on health	−0.465	**<0.001**	−0.439	**<0.001**

Data are presented as means (standard deviation) or Pearson correlation coefficient. In bold, informal caregiver outcomes significantly (*p* < 0.05) associated with informal caregivers’ quality of life. ^†^ Association between informal caregiver outcomes and EQ-5D. ANOVA test was used for categorical outcomes and correlation test for continuous outcomes. ^‡^ Association between informal caregiver outcomes and VAS. ANOVA test was used for categorical outcomes and correlation test for continuous outcomes. EQ-5D: European Quality of Life 5-Dimension scale, VAS: Visual Analogue Scale, ZBI: Zarit Burden Interviews, GHQ-12: General Health Questionnaire (12-item version), FCI: Family Caregiving Inventory, FIN: Family Inventory of Needs, PCSS: Pearlin Caregivers’ Stress Scale, KESO: Knowledge Expectations of Significant Other scale, RKSO: Received Knowledge of Significant Other scale, CRA: Caregiver Reaction Assessment.

**Table 5:** Multiple lineal regression model for informal caregivers’ quality of life.

Parameter	β Coefficient	Std. Error	t Value	Sig.	VIF
**Quality of Life (EQ-5D)**					
Intercept	0.847	0.033	25.340	<0.001	
PwD gender, female	0.078	0.022	3.486	0.001	1.02
Caregiver burden (ZBI)	−0.003	0.001	−3.225	0.002	1.58
Impact on health (CRA)	−0.010	0.003	−3.042	0.003	1.59
Adjusted R2 = 0.318					
**Self-reported quality of life (VAS)**					
Intercept	95.365	4.029	23.669	<0.001	
Time since diagnosis, years	−1.049	0.423	−2.480	0.015	1.02
Psychological wellbeing (GHQ-12)	−0.869	0.547	−1.589	0.115	2.03
Impact on health (CRA)	−1.337	0.421	−3.178	0.002	2.07
Adjusted R2 = 0.302					

EQ-5D: European Quality of Life 5-Dimension scale, VAS: Visual Analogue Scale, ZBI: Zarit Burden Interviews, GHQ-12: General Health Questionnaire (12-item version), CRA: Caregiver Reaction Assessment., Std.: Standard, Sig.: Significance, VIF: Variance Inflation Factor.

## References

[1] Gale S.A., Acar A., Daffner K.R. (2018). Dementia. Am. J. Med..

[2] Ponjoan A., Garre-Olmo J., Blanch J., Fages E., Alves-Cabratosa L., Martí-Lluch R., Comas-Cufí M., Parramon D., Garcia-Gil M., Ramos R. (2019). Epidemiology of dementia: Prevalence and incidence estimates using validated electronic health records from primary care. Clin. Epidemiol..

[3] Alzheimer Cataluña Fundació Lo Que no Sabéis sobre las Demencias y el Alzheimer. https://alzheimercatalunya.org/es/demencias-y-alzheimer/.

[4] Rose K.M., Williams I.C., Anderson J.G., Geldmacher D.S. (2020). Development and Validation of the Family Quality of Life in Dementia Scale. Gerontology.

[5] Frias C.E., Garcia-Pascual M., Montoro M., Ribas N., Risco E., Zabalegui A. (2020). Effectiveness of a Psychoeducational Intervention for Caregivers of People with Dementia with Regard to Burden, Anxiety and Depression: A Systematic Review. J. Adv. Nurs..

[6] Liu Z., Sun Y.Y., Zhong B.L. (2018). Mindfulness-based stress reduction for family carers of people with dementia. Cochrane Database Syst. Rev..

[7] Seitz D., Chan C.C., Newton H.T., Gill S.S., Herrmann N., Smailagic N., Nikolaou V., Fage B.A. (2018). Mini-Cog for the diagnosis of Alzheimer’s disease dementia and other dementias within a primary care setting. Cochrane Database Syst. Rev..

[8] Balash Y., Korczyn A.D., Knaani J., Migirov A.A., Gurevich T. (2017). Quality-of-life perception by Parkinson’s disease patients and caregivers. Acta Neurol. Scand..

[9] Miguel S., Alvira M., Farré M., Risco E., Cabrera E., Zabalegui A. (2016). Quality of life and associated factors in older people with dementia living in long-term institutional care and home care. Eur. Geriatr. Med..

[10] The EuroQol Group (1990). EuroQol—A new facility for the measurement of health-related quality of life. Health Policy.

[11] Martín-Carrasco M., Salvadó I., Nadal S., Miji L.C., Rico J.M., Lanz P., Taussig M.I. (1996). Adaptación para nuestro medio de la Escala de Sobrecarga del Cuidador (Caregiver Burden Interview) de Zarit. Rev. Gerontol..

[12] Romppel M., Braehler E., Roth M., Glaesmer H. (2013). What is the General Health Questionnaire-12 assessing? Dimensionality and psychometric properties of the General Health Questionnaire-12 in a large scale German population sample. Compr. Psychiatry.

[13] Rocha K.B., Pérez K., Rodríguez-Sanz M., Borrel C., Obiols J.E. (2011). Propiedades psicométricas y valores normativos del General Health Questionnaire (GHQ-12) en población general española. Int. J. Clin. Health Psychol..

[14] Alvira M.C., Risco E., Cabrera E., Farré M., Hallberg I.R., Bleijlevens M.H.C., Meyer G., Koskenniemi J., López M.E.S., Zabalegui A. (2015). The association between positive-negative reactions of informal caregivers of people with dementia and health outcomes in eight European countries: A cross-sectional study. J. Adv. Nurs..

[15] Alvira C., Cabrera E., Kostov B., Risco E., Farré M., Miguel S., Zabalegui A. (2020). Validity and reliability of the Spanish caregiver reaction assessment scale for caregivers of people with dementia. Int. J. Nurs. Pr..

[16] Folstein M., Folstein S., McHugh P. (1975). “Mini-mental state”. A practical method for grading the cognitive state of patients for the clinician. J. Psychiatry Res..

[17] Reisberg B., Ferris S.H., De Leon M.J., Crook T. (1982). The Global Deterioration Scale for assessment of primary degenerative dementia. Am. J. Psychiatry.

[18] Gómez-Gallego M., Gómez-Amor J., Gómez-García J. (2012). Validation of the Spanish version of the QoL-AD scale in Alzheimer disease patients, their carers, and health professionals. Neurología.

[19] Vilalta-Franch J., Lozano-Gallego M., Hernández-Ferrándiz M., Llinàs-Reglà J., López-Pousa S., López O.L. (1999). The Neuropsychiatric Inventory. Psychometric properties of its adaptation into Spanish. Rev. Neurol..

[20] Yamashita T., Yamashita K., Kamimura R. (2007). A Stepwise AIC Method for Variable Selection in Linear Regression. Commun. Stat. Theory Methods.

[21] Sheather S. (2009). A Modern Approach to Regression with R.

[22] R Core Team (2018). R: A Language and Environment for Statistical Computing.

[23] Gonyea J.G., López L.M., Velásquez E.H. (2014). The effectiveness of a culturally sensitive cognitive behavioural group intervention for Latino Alzheimer’s Caregivers. Gerontologist.

[24] Tang S.-H., Chio O.-I., Chang L.-H., Mao H.-F., Chen L.-H., Yip P.-K., Hwang J.-P. (2018). Caregiver active participation in psychoeducational intervention improved caregiving skills and competency. Geriatr. Gerontol. Int..

[25] Kerpershoek L., De Vugt M., Wolfs C., Woods B., Jelley H., Orrell M., Stephan A., Bieber A., Meyer G., Selbaek G. (2018). Needs and quality of life of people with middle-stage dementia and their family carers from the European Actifcare study. When informal care alone may not suffice. Aging Ment. Heal..

[26] Karg N., Graessel E., Randzio O., Pendergrass A. (2018). Dementia as a predictor of care-related quality of life in informal caregivers: A cross-sectional study to investigate differences in health-related outcomes between dementia and non-dementia caregivers. BMC Geriatr..

[27] Lee M., Ryoo J.H., Crowder J., Byon H.D., Wiiliams I.C. (2020). A systematic review and meta-analysis on effective interventions for health-related quality of life among caregivers of people with dementia. J. Adv. Nurs..

[28] Kaizik C., Caga J., Camino J., O’Connor C.M.C., McKinnon C., Oyebode J.R., Piguet O., Hodges J.R., Mioshi E. (2017). Factors Underpinning Caregiver Burden in Frontotemporal Dementia Differ in Spouses and their Children. J. Alzheimer Dis..

[29] Acosta D., Rottbeck R., Rodríguez G., Ferri C., Prince M.J. (2008). The epidemiology of dependency among urban-dwelling older people in the Dominican Republic; a cross-sectional survey. BMC Public Heal..

[30] Farina N., Page T.E., Daley S., Brown A., Bowling A., Basset T., Livingston G., Knapp M., Murray J., Banerjee S. (2017). Factors associated with the quality of life of family carers of people with dementia: A systematic review. Alzheimer Dement..

[31] Cruz M.R.S., Hidalgo P.C., Lee M.S., Thomas C.W., Holroyd S. (2017). Buspirone for the treatment of dementia with behavioral disturbance. Int. Psychogeriatrics.

[32] Quinn C., Nelis S.M., Martyr A., Victor C., Morris R.G., Clare L., IDEAL Study Team (2019). Influence of positive and negative dimensions of dementia caregiving on caregiver well-being and satisfaction with life: Findings from the IDEAL study. Am. J. Geriatr. Psychiatry.

[33] Sun X., Ge J., Meng H., Chen Z., Liu D. (2019). The Influence of Social Support and Care Burden on Depression among Caregivers of Patients with Severe Mental Illness in Rural Areas of Sichuan, China. Int. J. Environ. Res. Public Health.

